# **“**D2 plus” lymphadenectomy is associated with improved survival in distal gastric cancer with clinical serosa invasion: a propensity score analysis

**DOI:** 10.1038/s41598-019-55535-7

**Published:** 2019-12-16

**Authors:** Yuexiang Liang, Jingli Cui, Yaoqing Cai, Lijie Liu, Jianghao Zhou, Qiang Li, Junmei Wu, Donglei He

**Affiliations:** 1 Department of gastrointestinal oncology, The First Affiliated Hospital of Hainan Medical University, Longhua Road, Longhua District, Haikou City, 570100 Hainan Province China; 2 Department of general surgery, Weifang People’s Hospital, Guangwen street, Kuiwen District, Weifang City, 261000 Shandong Province China; 3 Department of gastrointestinal surgery, Hainan Cancer Hospital, Changbinxi Road, Xiuying District, Haikou City, 570102 Hainan Province China

**Keywords:** Gastric cancer, Outcomes research, Surgical oncology

## Abstract

The aim of this study was to elucidate the potential impact of “D2 plus” lymphadenectomy on the long-term survival of distal gastric cancer (GC) patients with clinical serosa invasion. A total of 394 distal GC patients with clinical serosa invasion who underwent at least standard D2 lymphadenectomy were enrolled. Patients were categorized into two groups according to the extent of lymphadenectomy: D2 group and “D2 plus” group. Propensity score matching was used to adjust for the differences in baseline characteristics. In the multivariate analysis for the whole study series, extent of lymphadenectomy was an independent prognostic factor for GC patients (*P* = 0.011). With the strata analysis, the significant prognostic differences between the two groups were only observed in patients at the IIIa-b or N1-3a stages. After matching, patients in “D2 plus” group still demonstrated a significantly higher 5-year overall survival rate than those in D2 group (55.3% versus 43.9%, *P* = 0.042). The common therapeutic value index of No. 12b, No. 12p, No. 14v and No. 13 LNs was 4.6, which was close to that of No. 5 LN station. In conclusion, “D2 plus” lymphadenectomy may be associated with improved overall survival in distal GC with clinical serosa invasion.

## Introduction

Standard treatment for gastric cancer (GC) according to tumor stage has been established in the Japanese GC treatment guidelines. Curative gastrectomy plus D2 lymph node (LN) dissection has been regarded as the standard surgery for potentially curable T2-4 tumors as well as cT1N^+^ tumors^1^. Though more extended LN dissection beyond D2 range is considered as non-standard surgical procedure, its clinical significance has been evaluated in several studies^2–11^. Masuda *et al*.^2^ found that No. 14v LN (14v) dissection was associated with improved overall survival (OS) rate for GC patients with 14v metastasis but without para-aortic LN metastasis. Liang *et al*.^3^ demonstrated that D2 plus 14v dissection could bring survival benefits for distal GC staged TNM IIIb-c comparing to standard D2 dissection. It was reported that No. 13v LN was often involved in GC with duodenum invasion^4,12^. Previous studies used therapeutic value index (TVI) to evaluate the value of LN dissection and confirmed that TVI of No. 13 LN was equivalent to that of second-tier LNs in distal GC with duodenum invasion^4,12^. Feng *et al*.^5^ demonstrated that the metastatic rate of No. 12p (12p) and No. 12b (12b) LNs were 9.2% and 3.1%, respectively. Even after curative resection, the 5-year OS rate was significantly lesser for GC patients with 12b or 12p LNs metastases than those without (13.3% versus 35.1%, *P* = 0.022). These studies specially focused on a single LN station such as 12b, 12p, 14v or No. 13 LN, and the prognostic value of dissection of multiple LNs beyond D2 range was rarely evaluated. However, these LNs were usually removed together in extended lymphadenectomy. For example, in advanced distal GC with duodenum invasion, the metastatic rates of 14v and No. 13 LNs are relatively high, and hepatoduodenal ligament LNs are often involved. In general, LNs including 12b, 12p, 14v and No. 13 LN are simultaneously dissected in “D2 plus” lymphadenectomy.

In this study, we particularly focused on distal GC with clinical serosa invasion, which was at higher risk of LNs metastases beyond D2 range. The aim of this study was to elucidate the prognostic value of “D2 plus” lymphadenectomy including 12b, 12p, 14v and No. 13 LN dissection in distal GC patients with clinical serosa invasion after curative surgery by means of multivariate Cox regression and propensity score matching analyses.

## Materials and Methods

### Patients

**Figure 1:**
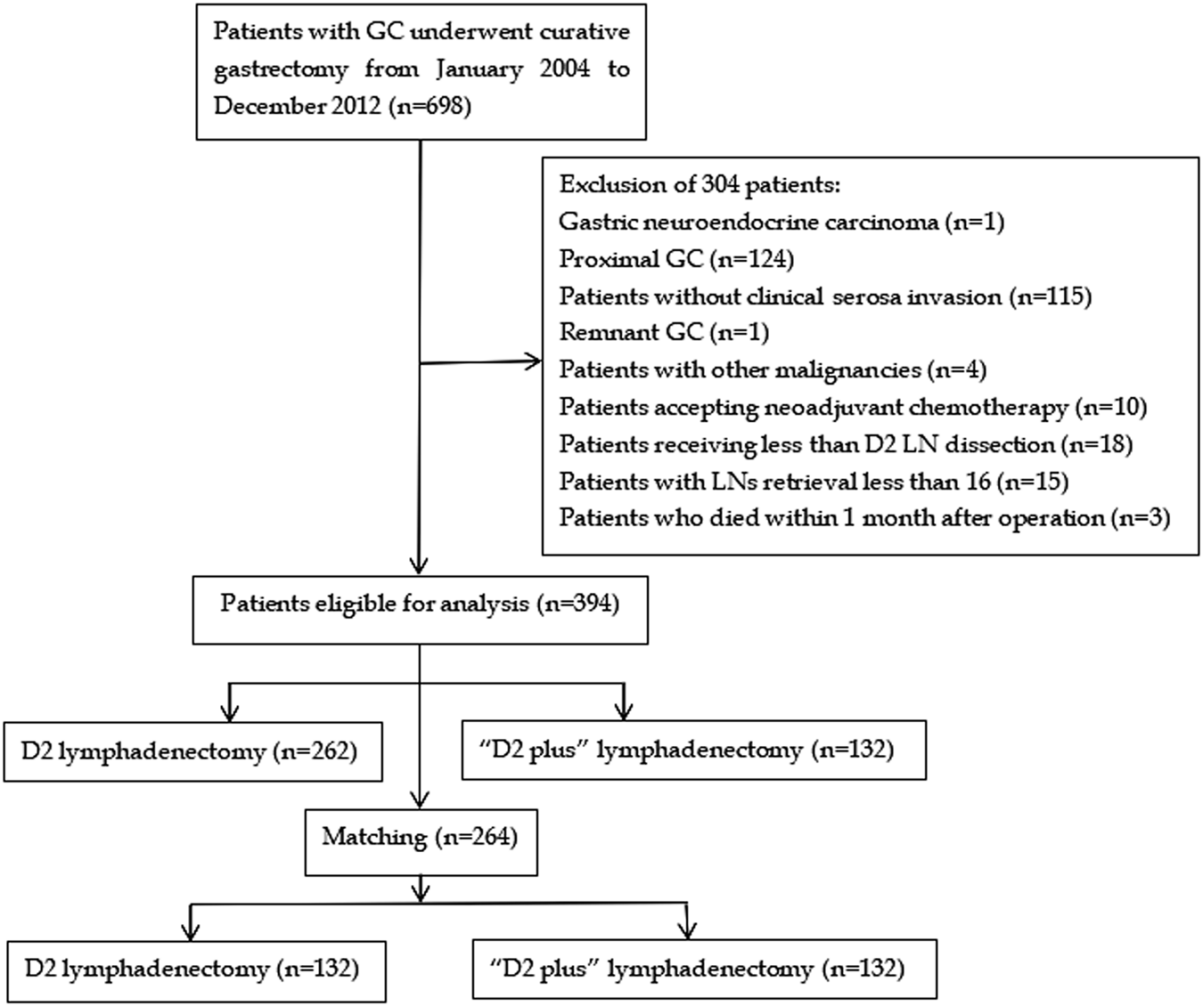
The criteria for inclusion and exclusion of all patients.

This study was reviewed and approved by the Ethics Committee of the First Affiliated Hospital of Hainan Medical University. All the patients signed an informed consent form for the operation including surgical procedure. All processes involved in this study were in accordance with the standards of the institutional Ethics Committee. From January 2004 to December 2012, 698 patients with GC who underwent curative gastrectomy at the First Affiliated Hospital of Hainan Medical University were eligible for this study. The flow chart and exclusion criteria of this study were shown in Fig. 1. After exclusion of 304 patients, ultimately, 394 patients were enrolled in this study.

As for preoperative staging, it was performed by means of enhanced computed tomography (CT) scan or endoscopic ultrasonography (EUS). Indications of T4a included loss of the bright line recognized as serosa in EUS and disappearance of perigastric fat layer in enhanced CT scan. And indication of T4b was extension of the mass into surrounding organs in EUS or CT scan. Patients were categorized into two groups according to the extent of lymphadenectomy: D2 group, including those receiving standard D2 LNs dissection; and “D2 plus” group, composed of those undergoing D2 plus 12b, 12p, 14v and No. 13 LNs dissection. As a result, 262 patients were assigned to D2 group and 132 patients to “D2 plus” group.

### Evaluation of clinicopathological variables and survival

Clinicopathological factors studied were as follows: gender, age, tumor location, tumor diameter, Borrmann type, histological type, T stage, N stage, metastatic LN ratio (rN), TNM stage, extent of lymphadenectomy, type of gastrectomy, total number of LNs retrieval, extranodal tumor deposits and postoperative chemotherapy. Because of the differences in the extent of lymphadenectomy and the count of LNs retrieval between the two groups, which might lead to stage migration, we used rN to eliminate it in propensity score analysis. In previous studies^13,14^, rN was confirmed to be a good compensation for stage migration which was caused by the different extent and number of LNs dissection. By using the log-rank test, 0.2 and 0.5 were identified as the best thresholds of rN in the present study (rN0 = 0, 0 < rN1 ≤ 0.2, 0.2 < rN2 ≤ 0.5, rN3 > 0.5).

Sasako *et al*.^15^ proposed that the TVI could be used to evaluate the clinical value of LN dissection. In their study, the TVI was calculated by multiplying the metastatic rate of the station by the 5-year OS rate of patients with metastasis to that LN station. In this study, we also calculated the TVI of each LN station to assess the necessity of LN dissection.

Tumor staging was in accordance with the eighth edition of the Union for International Cancer Control (UICC) TNM classification system, whereas extent of lymphadenectomy and LN stations were defined according to the fourth English Edition of the Japanese Gastric Cancer Treatment Guidelines and the third English Edition of the Japanese Classification of Gastric Carcinoma^16^. The tumors were categorized into two types according to histology: (1) differentiated type, including well or moderately differentiated and papillary adenocarcinoma; (2) undifferentiated type, including signet ring cell carcinoma, mucinous carcinoma, and poorly differentiated or undifferentiated adenocarcinoma.

### Follow up

The follow-up of the patients was carried out by the research nurse of our department. Patients were followed-up every three months for up to two years, then every six months for three to five years, and then every year or until death. Physical examination, laboratory tests (including CEA and CA19-9), and abdominal Doppler ultrasound were required at each visit, while chest and abdominal enhanced computed tomography scans were performed every 6 months or each year. Gastroscopy was obtained every year. The OS was calculated from the time of operation to the time of death or final follow-up. The date of the final follow-up was December 31, 2017.

### Statistical analysis

The continuous variables were analyzed by means of the Student’s t test. The categorical variables were analyzed using the Chi-square or Fisher exact test. The OS curves were calculated using the Kaplan-Meier method based on the duration of time between the primary surgical treatment and the final follow-up or death. The log-rank test was used to evaluate the significant differences between curves. The Cox proportional hazards regression model was implemented to determine the independent prognostic factors. In order to overcome the deviation caused by the different distribution of covariates in the two groups, the propensity score analysis was applied to get a one-to-one match by using the nearest-neighbor matching method. And we imposed a caliper of 0.25 of the standard deviation (SD) of the logit of the propensity score. Factors unrelated to the extent of lymphadenectomy were included in the propensity model. P < 0.050 (bilateral) was considered to be statistically significant. The statistical analysis was accomplished by using the statistical analysis program package SPSS 22.0 (IBM Corporation, NY, USA).

## Results

### Clinicopathologic features and survival of the whole study series

The median follow-up was 49 (range: 1–115) months. Of the 394 patients, 280 were male (71.1%), and 114 were female (28.9%). The age ranged from 27 to 81 years old, with a median age of 61 years. Of the 394 GC patients with curative resection, 165 patients had total gastrectomy, and 229 patients underwent subtotal gastrectomy. Among them, 296 patients accepted postoperative adjuvant chemotherapy with FOLFOX6, XELOX, S-1 or capecitabine.

Patients were classified into two groups based on the extent of lymphadenectomy. Table 1 displays the status and number of LNs harvested in both groups. The total number of LNs harvested in “D2 plus” group was higher than that in D2 group, while there were no significant difference in the number of LNs harvested at other regional stations (No. 1–12a stations) between the two groups. The number of metastatic LNs in “D2 plus” group was similar to that in D2 group as well. Other clinicopathologic variables were compared in Table 2. There was no significant difference in Borrmann type, histological type, N stage, TNM stage, extranodal tumor deposits, type of gastrectomy and postoperative chemotherapy between the two groups. Compared with “D2 plus” group, the proportion of male patients in D2 group was larger (75.6% versus 63.6%, *P* = 0.021), the mean age was elder (62.3 ± 10.8 versus 56.7 ± 12.0, *P* < 0.001), the ratio of T3 (12.2% versus 3.9%) and rN3 (16.8% versus 5.3%) stage disease was higher, but the percentage of tumors located at distal stomach was smaller (22.1% versus 40.9%).

**Table 1:** Status and number of lymph nodes harvested from this cohort as a function of extent of lymph node dissection.

	D2 (n = 262)	D2 plus (n = 132)	*P*
Total number LNs dissected	26.16 (25.07–27.25)	32.63 (30.57–34.69)	<0.001
Total number of LNs metastases	5.70 (4.60–6.80)	4.70 (3.68–5.74)	0.248
Number of 12b, 12p, 13 and 14v LNs metastases	NA	0.30 (0.14–0.47)	NA
Number of 12b, 12p, 13 and 14v LNs dissected	NA	4.99 (4.26–5.72)	NA
Number of LN 1-12a metastases	5.70 (4.60–6.80)	4.42 (3.45–5.40)	0.087
Number of 1-12a LNs dissected	26.16 (25.07–27.25)	27.64 (25.92–29.36)	0.153

LN, lymph node.

**Table 2:** *C*linicopathological features of GC patients who underwent gastrectomy with curative intent grouped by the extent of lymph node dissection: data are reported for the whole study series and for one-to-one propensity-score matched pairs.

Characteristics	Whole study series			Matched pairs (Case-control Method)		
	D2(n = 262)	D2 plus(n = 132)	*P*	D2(n = 132)	D2 plus(n = 132)	*P*
Gender			0.021			0.189
Male/Female	198/66	84/48		94/38	84/48	
Age (yr)			<0.001			0.111
Mean ± sd	62.3 ± 10.8	56.7 ± 12.0		59.0 ± 10.5	56.7 ± 12.0	
Tumor location			<0.001			0.072
L/M/whole	58/170/34	54/61/17		37/78/17	54/61/17	
Borrmann type			0.436			0.218
I/II/III/IV	24/74/150/14	9/47/68/8		13/33/80/6	9/47/68/8	
Tumor diameter			0.590			0.799
<5 cm/≥5 cm	92/170	50/82		48/84	50/82	
Histology			0.092			0.455
Differentiated/Undifferentiated	72/190	26/106		31/101	26/106	
Depth of invasion			0.022			0.184
pT3/pT4a/pT4b	32/222/8	5/121/6		12/116/4	5/121/6	
N stage			0.651			0.801
pN0/pN1/pN2/pN3a/pN3b	80/36/76/48/22	38/26/37/21/10		37/22/46/19/8	38/26/37/21/10	
rN stage			0.003			0.321
rN0/rN1/rN2/rN3	82/78/58/44	38/58/29/7		38/50/29/15	38/58/29/7	
TNM stage			0.516			0.875
II/IIIa/IIIb/IIIc	88/98/52/24	37/59/26/10		37/65/21/9	37/59/26/10	
Average lymph nodes retrieval			<0.001			0.255
Mean ± sd	26.2 ± 8.9	32.6 ± 12.0		31.2 ± 7.9	32.6 ± 12.0	
Extranodal tumor deposits			0.788			0.682
Present/Absent	74/188	39/93		36/96	39/93	
Types of gastrectomy			0.355			0.213
Total/Subtotal	114/148	51/81		61/71	51/81	
Postoperative chemotherapy			0.484			0.394
Yes/No	194/68	102/30		96/36	102/30	

L, lower 1/3; M, middle 1/3; whole, both the lower and middle or more.

**Figure 2:**
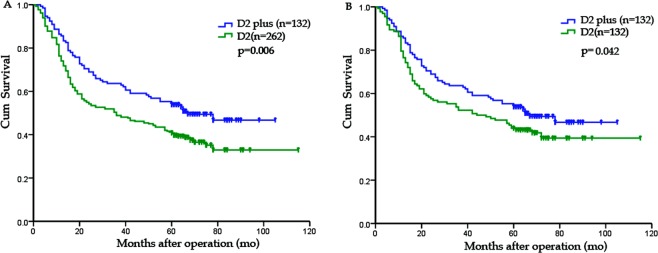
Prognosis of GC patients who underwent curative surgery. Patients were categorized into two groups according to the extent of lymphadenectomy: “D2 plus” group and D2 group. (**A**) Survival curve for all patients: the 5-year OS rates were 55.3% and 41.2% for “D2plus” group and D2 group, respectively (P = 0.006). (**B)** Survival curve for matched patients: the 5-year OS rates were 55.3% and 43.9% for “D2 plus” group and D2 group, respectively (P = 0.042).

In the whole study population, the 5-year OS rate of “D2 plus” group was significantly higher than that of D2 group (55.3% versus 41.2%, *P* = 0.006) (Fig. 2A). In the univariate analysis, the following nine factors had a significant impact on OS: age (<70 versus ≥70), tumor location, tumor diameter (<5 versus ≥5 cm), Borrmann type, TNM stage, type of gastrectomy, total number of LN retrieval (≥25 versus <25), extent of lymphadenectomy (D2 versus D2 plus) and extranodal tumor deposits (Table 3). Multivariate analysis confirmed that the extent of lymphadenectomy (hazard ratio was 0.658 for “D2 plus”, 95% CI, 0.476–0.909, *P* = 0.011) was an independent prognostic factor, as were the following: age (≥70), Borrmann type, TNM stage, type of gastrectomy, total number of LN retrieval and extranodal tumor deposits. Strata analysis revealed that “D2 plus” lymphadenectomy could contribute to improved OS in patients at III_a-b_ or N_1-3a_ stages, compared with D2 dissection (Table 4).

**Table 3:** The univariate and multivariate survival analyses of GC patients in the whole study series.

Characteristics	n	5-year OS (%)	Univariate analysis		Multivariate analysis	
			HR(95%CI)	*P*	HR(95%CI)	*P*
Gender						
Male	280	45.0	1 (ref)			
Female	114	48.2	0.926 (0.694–1.236)	0.604		
Age (yr)						
<70	301	49.8	1 (ref)		1 (ref)	
≥70	93	33.3	1.413 (1.058–1.888)	0.019	1.408 (1.025–1.933)	0.035
Tumor location						
Lower one-third	112	56.3	1 (ref)		1 (ref)	
Middle one-third	231	42.9	1.509 (1.106–2.060)	0.009	1.045 (0.740–1.447)	0.802
2/3 or more	51	37.3	1.485 (0.960–2.297)	0.076	1.168 (0.918–1.314)	0.056
Tumor diameter						
<5 cm	142	57.0	1 (ref)		1 (ref)	
≥5 cm	252	39.7	1.666 (1.255–2.213)	<0.001	1.034 (0.752–1.421)	0.838
Borrmann type						
I	33	78.8	1 (ref)		1 (ref)	
II	121	49.6	2.630 (1.311–5.277)	0.007	2.342 (1.141–4.805)	0.020
III	218	41.3	3.336 (1.697–6.557)	<0.001	2.589 (1.282–5.231)	0.008
IV	22	22.7	5.202 (2.310–11.714)	<0.001	4.176 (1.679–10.389)	0.002
Histology						
Differentiated	98	49.0	1 (ref)			
Undifferentiated	296	44.9	1.048 (0.777–1.413)	0.760		
TNM stage						
II	125	70.4	1 (ref)		1 (ref)	
IIIa	157	44.6	1.967 (1.387–2.791)	<0.001	1.707 (1.167–2.497)	0.006
IIIb	78	24.4	3.451 (2.359–5.050)	<0.001	3.685 (2.447–5.550)	<0.001
IIIc	34	11.8	6.321 (3.978–10.042)	<0.001	5.112 (2.984–8.759)	<0.001
Type of gastrectomy						
Subtotal	229	50.7	1 (ref)		1 (ref)	
Total	165	39.4	1.419 (1.095–1.838)	0.008	1.702 (1.226–2.363)	0.002
Extent of lymphadenectomy						
D2	262	41.2	1 (ref)		1 (ref)	
D2 plus	132	55.3	0.674 (0.507–0.897)	0.007	0.658 (0.476–0.909)	0.011
Total number of lymph node retrieval						
≥25	179	52.0	1 (ref)		1 (ref)	
<25	215	40.9	1.372 (1.053–1.787)	0.019	1.504 (1.092–2.072)	0.012
Extranodal tumor deposits						
Absent	281	52.7	1 (ref)		1 (ref)	
Present	113	29.2	2.110 (1.614–2.758)	<0.001	1.605 (1.200–2.147)	0.001
Postoperative chemotherapy						
Yes	296	48.6	1 (ref)			
No	98	37.8	1.221 (0.913–1.631)	0.178

**Table 4:** Strata survival analysis of the GC patients according to the extent of lymphadenectomy: data are reported for the whole study series and for matched pairs.

Tumor stage	5-year OS for whole study series (%)			5-year OS for Matched pairs (%)		
	D2	D2 plus	*P*	D2	D2 plus	*P*
N stage						
pN0	70.0	68.4	0.562	75.7	68.4	0.197
pN1	41.7	65.4	0.025	46.4	65.4	0.026
pN2	35.5	51.4	0.039	37.0	51.4	0.041
pN3a	16.7	42.9	0.006	21.1	42.9	0.046
pN3b	9.1	20.0	0.668	12.5	20.0	0.846
TNM stage						
II	70.5	70.3	0.766	74.4	70.3	0.278
IIIa	36.7	57.6	0.011	48.1	57.6	0.030
IIIb	15.4	42.3	0.001	19.0	42.3	0.020
IIIc	8.3	20.0	0.722	11.1	20.0	0.859

### Characteristics and prognosis of matched pairs

We selected 132 patients form the D2 group for one-to-one matching with the “D2 plus” group by using propensity scores. The median follow-up was 59 (range: 1–115) months. Patients characteristics after matching were shown in the right column of Table 2. Of the 262 patients in the D2 group, 132 cases were matched with the 132 patients of the “D2 plus” group after the adjustment of the covariates. All covariates were evenly distributed in the two matching groups. Following factors of matched patients in D2 group were similar to that of “D2 plus” group: gender, mean age, tumor location, tumor diameter, Borrmann type, histological type, T stage, N stage, rN stage, TNM stage, type of gastrectomy, average LNs retrieval, extranodal tumor deposits and postoperative chemotherapy.

After matching, patients of “D2 plus” group still demonstrated a significantly better OS that those of D2 group (5-year OS rate: 55.3% versus 43.9%, *P* = 0.042) (Fig. 2B). With the strata analysis, the 5-year OS rate of “D2 plus” group was significantly higher that that of D2 group at III_a-b_ or N_1-3a_ stages (Table 4). In the multivariate analysis, extent of lymphadenectomy (HR was 0.697 for “D2 plus”, 95% CI, 0.489–0.993, *P* = 0.046) remained an independent prognostic factor, as were age, Borrmann type, extranodal tumor deposits, type of gastrectomy and TNM stage (Table 5).

**Table 5:** Multivariate survival analysis of matched GC patients.

Factors		*P*	Hazard ratio	95%CI
Age(yrs)	<70/≥70	0.011	1.664	1.126–2.458
Tumor location	Middle one-third/Lower one-third	0.546	1.139	0.747–1.737
	2/3 or more/Lower one-third	0.240	1.373	0.809–2.328
Tumor diameter	≥5 cm/<5 cm	0.267	1.245	0.845–1.834
Borrmann type	Type II/type I	0.019	3.097	1.209–7.937
	Type III/type I	0.024	2.898	1.153–7.286
	Type IV/type I	0.006	4.758	1.548–14.623
Type of gastrectomy	Total/subtotal	0.028	1.584	1.050–2.389
Extronodal tumor deposits	Present/absent	0.003	1.714	1.197–2.453
Extent of lymphadenectomy	D2 plus/D2	0.046	0.697	0.489–0.993
TNM stage	IIIa/II	0.026	1.745	1.069–2.846
	IIIb/II	<0.001	3.082	1.752–5.423
	IIIc/II	<0.001	4.158	2.128–8.123

### TVI of LNs dissection

The TVI of each LN station was shown in Table 6. The 5-year OS rate in 12b metastasis group was 22.2%, that in 12p metastasis group was 25.0%, that in No. 13 LN metastasis group was 42.9%, and that in 14v metastasis group was 33.3%. The metastatic rate of 12b (6.8%), 12p (3.0%) and No. 13 LN (5.3%) was lower than that of 14v (11.5%). The TVI of 12b, 12p, No. 13 LN and 14v were 1.5, 0.75, 2.3 and 3.9, respectively. The common TVI of LN stations beyond D2 range (including 12b, 12p, No. 13 LN and 14v) was 4.6, similar to that of No. 2 (4.2) and No. 12a (4.1) LN stations, but greater than that of No. 4sa, No. 10, No. 11p, and No. 11d LN stations.

**Table 6:** Therapeutic value index of each regional lymph node station.

LNs station number	Metastatic rate (%)	5-year OS (%)	Therapeutic value index(%)
1	18.3 (72/394)	37.5	6.9
2	13.3(22/165)	31.8	4.2
3	42.1(166/394)	37.3	15.7
4sa	8.5(14/165)	28.6	2.4
4sb	19.0 (75/394)	33.3	6.3
4d	24.9 (98/394)	35.7	8.9
5	16.8 (66/394)	33.3	5.6
6	26.9(106/394)	26.4	7.1
7	22.3(88/394)	33.0	7.4
8a	17.8 (70/394)	34.3	6.1
9	18.5 (73/394)	38.4	7.1
10	9.3 (17/183)	35.3	3.3
11p	11.2 (44/394)	29.5	3.3
11d	8.2(23/282)	26.1	2.1
12a	13.2 (52/394)	30.8	4.1
12b	6.8(9/132)	22.2	1.5
12p	3.0(4/132)	25.0	0.75
13	5.3(7/132)	42.9	2.3
14v	11.4(15/132)	33.3	3.9
12b + 12p + 13 + 14v	15.9(21/132)	28.6	4.6

**Figure 3:**
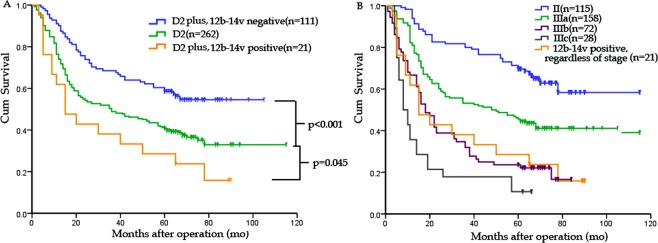
(**A**) Survival curve for GC patients categorized by extent of lymphadenectomy and status of 12b-14v LNs. Patients receiving “D2 plus” lymphadenectomy with 12b-14v LNs metastases had a significant lower OS rate than those undergoing D2 lymphadenectomy (28.6% versus 41.2%, P = 0.045). (**B**) Survival curves for GC patients categorized by TNM stage and 12b-14v LNs status. The 5-year OS was 28.6% for 12b-14v positive patients (regardless of TNM stage) and 23.6% for patients with IIIb stage disease, respectively. P = 0.874 (IIIb stage versus positive 12b-14v LNs regardless of the TNM stage, log-rank test).

A total of 21 patients with GC had LNs metastases beyond D2 range. The 5-year OS rate of these patients was significantly lower than that of D2 group (28.6% versus 41.2%, *P* = 0.045) (Fig. 3A). The OS rate of patients with LNs metastases beyond D2 range was similar to that of III_b_ stage disease (Fig. 3B).

### Recurrence data of the whole study series

As shown in Table 7, the overall recurrence rate of D2 group (69.5% versus 51.5%, *P* < 0.001), especially the recurrence rate of LNs (18.7% versus 2.3%, *P* < 0.001), was significantly higher than that of “D2 plus” group. There was no significant difference in other recurrence types between the two groups.

**Table 7:** Type of initial recurrence in the whole study series.

Type of recurrence	D2 group (n = 262)	D2 plus group (n = 132)	*P*
Locoregional	61 (23.3)	11 (8.3)	<0.001
Lymph node	49 (18.7)	3 (2.3)	<0.001
Gastric stump	4 (1.5)	4 (3.0)	0.318
Anastomosis	3 (1.1)	1 (0.8)	0.717
Gastric bed	5 (1.9)	3 (2.3)	0.809
Peritoneal	66 (25.2)	31 (23.5)	0.711
Hematogenous	37 (14.1)	20 (15.2)	0.784
Combined	18 (6.9)	6 (4.5)	0.362
Overall recurrence	182 (69.5)	68 (51.5)	<0.001

## Discussion

The extent of lymphadenectomy has always been a hot research point in surgical treatment of GC. It had been confirmed that LNs located in the hepatoduodenal ligament (including 12a, 12b and 12p), posterior of pancreatic head or root of superior mesenteric vein were often involved in distal GC with serosa invasion^12,17–19^. It was also reveled that “D2 plus” lymphadenectomy could obtain more LNs than standard D2 lymphadenectomy, and that increasing the number of LNs retrieval might contribute to adequate staging and better survival^20–23^. In addition, the morbidity and mortality of”D2 plus” lymphadenectomy were same as those of D2 lymphadenectomy^6^. Therefore, we conducted this study to investigate the effect of D2 or “D2 plus” lymphadenectomy on the OS of distal GC patients with clinical serosa invasion. We found that GC patients who underwent “D2 plus” lymphadenectomy had more LNs retrieval and improved OS than those who had D2 lymphadenectomy. And multivariate analysis confirmed that the extent of lymphadenectomy was an independent prognostic factor. Even after propensity score matching analysis, the 5-year OS rate of the “D2 plus” group was significantly higher than that of the D2 group (55.3% versus 43.9%, *P* = 0.042).

Previous study had focused on a single LN station and negated the value of LNs dissection because of its lower metastatic rate and poorer survival^4,5,15^. An *et al*.^18^ demonstrated that the prognosis of GC patients with 14v metastasis was significantly worse than that of patients without 14v metastasis, and the OS rate of patients with 14v metastasis was even lower than that of patients with stage IV disease. Therefore, they came to the conclusion that 14v should be excluded from loco-regional LNs. Feng *et al*.^5^ revealed that the metastatic rates of 12p and 12b were 9.2% and 3.1%, respectively, and GC patients with 12b and 12p metastases had a significant lesser OS rate than those without (13.3% versus 35.1%, *P* = 0.022). However, Kumagai *et al*.^4^ reported that the metastatic rates of 12b and 12p were 18.3% and 2.8% in GC patients with duodenal invasion, respectively, which were different from Feng’s results. The difference in metastatic rate may due to different inclusion criteria and diverse indications to extended LN dissection. Until now, there is no consensus on the metastatic rate of 12b and 12p.

In other studies, TVI was implemented to evaluate the actual benefits of LNs dissection^4,12^. Tokunaga *et al*.^12^ found that the TVI of dissection of No. 13 LN was 4.19, which was equal to that of the second-tier LNs, such as No. 9 and No. 11p LNs. Recently, a Japanese study^4^ specially focused on GC patients with duodenal invasion. The results reveled that the TVI of 14v and No. 13 LN were 6.1 and 6.8, respectively, which were equivalent to that of No. 9 (6.6) and No. 7 (5.3) LNs, while the TVI of 12b was 7.4, which was greater that that of No. 12a (3.3) and No. 5 (5.0) LNs. Because the TVIs of 12b, 14v and No. 13 LN were comparable to the TVI of the second-tier LNs in these studies, they came to the conclusion that dissection of 12b, 14v and No. 13 LN should be an option for distal GC with duodenal invasion. In this study, the TVI was calculated by multiplying the metastatic rate of LN by the 5-year OS rate of the patients with metastasis to that station. We found that the TVIs of 12b, 12p, No. 13 LN, and 14v were 1.5, 0.75, 2.3 and 3.9, respectively. Though the TVIs of 12b and 12p were lower, three patients with 12b or 12p metastasis still survived for more than five years after surgery. Meanwhile, the 5-year OS rate of patients with No. 13 LN metastasis was as high as 42.9%. These patients could indeed benefit form “D2 plus” lymphadenectomy. Our results also reveled the OS of patients with these LNs metastases beyond D2 range was similar to that of patients with staged III_b_ disease. We believe that although TVI was a reliable parameter to evaluate the significance of LN dissection, it could not completely reflect the benefits of LN dissection. So far, randomized controlled studies were still the golden standard to determine the necessity of LN dissection. This was confirmed in Eom’ study which evaluated the effects of D2 plus No. 13 LN dissection on OS of GC patients^24^. It was found that the metastatic rate of No. 13 LN was 6.7%, and the TVI was low. However, in the multivariate analysis, dissection of No. 13 LN was identified as an independent prognostic factor for distal GC clinically staged III/IV^24^. Actually, previous studies^3,25^ had confirmed the prognostic benefits of 14v dissection by retrospective randomized controlled studies. For example, Liang *et al*.^3^ reveled that addition of 14v to D2 lymphadenectomy could contribute to improved OS and reduced loco-regional LN recurrence rate in distal GC patients pathologically staged III_b_ and III_c_. Eom *et al*.^25^ demonstrated that 14v dissection was associated with higher OS rate of distal GC patients clinically staged III/IV.

As the necessity of dissection of these LNs beyond D2 range were still controversial, and most of these LNs were removed together in “D2 plus” lymphadenectomy, our study focused on the common therapeutic value of dissection of these LNs, rather than a single station. Considering the stage migration caused by different extent of lymphadenectomy and the distribution of covariates between the two groups, a one-to-one propensity score matching method and stratified analysis were applied. Our results reveled that the OS rate of distal GC patients with clinical serosa invasion who underwent “D2 plus” lymphadenectomy was significantly higher than that of patients who received standard D2 lymphadenectomy, and this trend still existed after matching. Both pre- and post-matching stratified analyses revealed that “D2 plus” LN dissection could contribute to improved OS in patients at III_a-b_ or N_1-3a_ stages, compared with D2 dissection. The common TVI of these LNs including 12b, 12p, No. 13 LN and 14v was 4.6, close to that of No. 5 LN, but greater than that of the second-tier LNs, such as No. 12a, and No. 11p LNs. From this point of view, it was reasonable to remove these LNs together. In fact, the metastatic rate of LNs beyond D2 range was 15.9% in this study. Theoretically, extended “D2 plus” lymphadenectomy could decrease the residual of the positive LNs and thus reduce the recurrence rate. Our study confirmed that overall recurrence rate of the “D2 plus” group, especially LNs recurrence was lower than that of the D2 group. This was consistent with the results of the previous studies^3,26^. We believe that distal GC with these LNs metastasis is at local advanced stage, rather than systemic disease. Removal of these LNs is helpful to increase the rate of curative resection and reduce the incidence of local recurrence, thus improving the OS rate.

Even so, we still need to pay attention to the limitations of this study. First of all, the nature of the retrospective and single-institutional design determined that the level of evidence in this study was low. Secondly, the sample of this study was a little small. Thirdly, there was no analysis of complications correlated with lymphadenectomy, such as blood loss, side injury and infection. Although it had been confirmed that the incidence of complications associated with “D2 plus” lymphadencetomy was substantially the same as that of standard D2 lymphadenectomy^6^, the complications of extended lymph node dissection should be noted, especially for non-specialized surgeons. Last but not least, though propensity score matching was able to eliminate the imbalance of baseline characteristics between the two groups, it could not overcome bias due to selective bias. For example, factors correlated with choice of performing D2 or “D2 plus” lymphadenectomy. Usually, “D2 plus” lymphadenectomy was more likely to be performed in patients with better physical condition and younger mean age, which may contribute to improved survival. Actually, age (≥70 year) was identified as an independent prognostic factor in previous study^27^. All in all, to overcome these limitations, multicenter prospective studies are needed.

## Conclusion

So far, as no prospective randomized controlled trial has been conducted to investigate the effect of “D2 plus” lymphadenectomy on the OS of patients with GC, and long-term survival patients with these LNs including 12b, 12p, 14v and No. 13 LN metastases are not uncommon. According the the results of this study and other retrospective studies, we concluded that the addition of 12b, 12p, 14v and No. 13 LN to D2 lymphadenectomy should be an option for distal GC with clinical serosa invasion.
